# Cyanidin-3-O-Glucoside Alleviates Hepatic Steatosis and Inflammation in High-Fat Diet-Induced Non-Alcoholic Fatty Liver Disease Mice via the AMPK/SIRT1/NF-κB Pathway

**DOI:** 10.3390/ijms27114698

**Published:** 2026-05-23

**Authors:** Xiping Liu, Wenya Li, Xiang Xu, Jichun Wang, Yuhang Liu, Yuxi Ma, Xin Su, Xiaoxi Shen, Yi Yang

**Affiliations:** 1 Department of Biotechnology, College of Life Sciences, Jilin Normal University, Siping 136000, China; 15903495754@163.com (X.L.); 15886073099@163.com (W.L.); xuxiang42657@163.com (X.X.); 15648822307@163.com (J.W.); 18004340882@163.com (Y.L.); 17614410885@163.com (Y.M.); 2 Development and Utilization of Wild Plant Resources in Changbai Mountain, Boda College, Jilin Normal University, Siping 136000, China; suxin97@163.com (X.S.); shenxiaoxijiankang@126.com (X.S.)

**Keywords:** Cyanidin-3-O-Glucoside, non-alcoholic fatty liver disease, lipid metabolism, AMPK pathway

## Abstract

Cyanidin-3-O-Glucoside (C3G) is the primary anthocyanin-active component in bilberry, exhibiting various pharmacological activities such as antioxidant, anti-inflammatory, and lipid metabolism-regulating effects. To address the clinical need for non-alcoholic fatty liver disease (NAFLD) prevention and treatment, this study aimed to investigate the ameliorative effects of C3G on NAFLD pathology and elucidate its molecular mechanisms of protection via the AMPK pathway. After a one-week acclimatization period, 20 six-week-old SPF mice were randomly divided into four groups: normal diet control (NCD), high-fat diet model (HFD), HFD + L-C3G (100 mg/kg/day), and HFD + H-C3G (200 mg/kg/day). Except for the NCD group, the remaining groups were fed a 60% high-fat diet for four weeks to establish an early-stage NAFLD model, with successful model construction verified by weight and liver weight gain. From the fifth week onward, C3G groups received daily administration for four consecutive weeks, while control groups were given an equal volume of distilled water. Liver function, lipid metabolism, oxidative stress, and inflammatory levels were assessed using ELISA, H&E staining, and other methods. The results showed that C3G restored liver function in NAFLD mice, improved lipid metabolism disorders, reduced oxidative stress and inflammatory responses, and alleviated liver pathological damage. Mechanistic studies revealed that C3G regulated the expression of mRNA and proteins related to the AMPK/SIRT1/NF-κB signaling pathway, activating the pathway by upregulating AMPK and its upstream regulators while inhibiting NF-κB-mediated inflammatory responses. This study confirmed that C3G can ameliorate high-fat diet-induced NAFLD lesions by activating the AMPK/SIRT1/NF-κB pathway, providing a potential intervention strategy for NAFLD prevention and treatment.

## 1. Introduction

In recent years, the global incidence of non-alcoholic fatty liver disease (NAFLD) has increased continuously due to sedentary lifestyles and unreasonable dietary structures. NAFLD, characterized by hepatic lipid deposition, has become one of the most common chronic liver diseases worldwide and places a heavy burden on public health. Therefore, it is particularly urgent to develop safe and effective intervention strategies from natural products [1,2]. NAFLD has become one of the chronic diseases that seriously threaten human health worldwide, mainly manifested as a clinical pathological syndrome characterized by hepatic cell steatosis and lipid storage [3,4]. NAFLD includes a range of diseases associated with excessive accumulation of liver lipids, from steatosis to non-alcoholic steatohepatitis, to cirrhosis and a series of complications such as portal hypertension, liver failure, and hepatocellular carcinoma [5]. At the pathophysiological level, the occurrence and progression of NAFLD are closely related to hepatic lipid metabolism disorders, cellular injury, inflammatory responses, and oxidative stress [6]. The Adenylate-Metabolizing Protein Kinase (AMPK) pathway plays a crucial role in regulating hepatic lipid metabolism. AMPK activation inhibits de novo lipid synthesis and promotes fatty acid oxidation, while its upstream regulator, Silencing Information Regulator 1 (SIRT1), further amplifies this effect by deacetylating key signaling molecules [7,8]. In addition, studies have found that sustained lipid overload activates the Nuclear Factor κB (NF-κB) inflammatory pathway, driving the progression of simple fatty liver to steatohepatitis [9]. However, there are currently no effective therapeutic drugs targeting this mechanism. Clinical treatment still relies mainly on conventional Western medicine and traditional Chinese medicine, but there are limitations, such as side effects or unclear mechanisms. Therefore, searching for safe and effective NAFLD prevention and treatment drugs from natural compounds has become a current research hotspot.

Among various natural resources, *Lonicera caerulea* L. (indigo fruit), as a characteristic northern crop, has attracted increasing attention due to its rich bioactive components and potential health benefits. The great advantage of *Lonicera caerulea* L. is its high resistance to low temperatures [10]. It is rich in various vitamins and mineral elements, and the Japanese indigenous people consider indigo fruit to be an elixir of life [11]. Indigo fruit contains abundant anthocyanins, which have a wide range of biological activities such as lipid-lowering, antioxidant, anti-inflammatory, anticancer, and liver protection [12,13,14]. Studies have shown that Cyanidin-3-O-Glucoside (C3G) is the main active ingredient in the anthocyanins of indigo fruit, accounting for 76.61–92% of the total anthocyanins [15]. Recent research on indigo fruit has confirmed that C3G in indigo fruit has protective effects on human health, including anti-aging and anti-obesity, reducing heart disease, and protecting the liver [16,17,18].

Based on the above background, we propose the core hypothesis of this study: C3G can improve lipid metabolism disorders and inhibit inflammatory responses in NAFLD mice by activating AMPK/SIRT1/NF-κB. Therefore, this study uses NAFLD mouse liver injury as a model to explore the improvement effect and potential molecular mechanism of C3G on NAFLD liver lipid metabolism disorder and inflammatory damage, aiming to provide a scientific basis for the prevention and treatment of NAFLD.

## 2. Results

### 2.1. Effects of C3G on Body Weight and Liver Mass in NAFLD Mice

**Figure 1:**
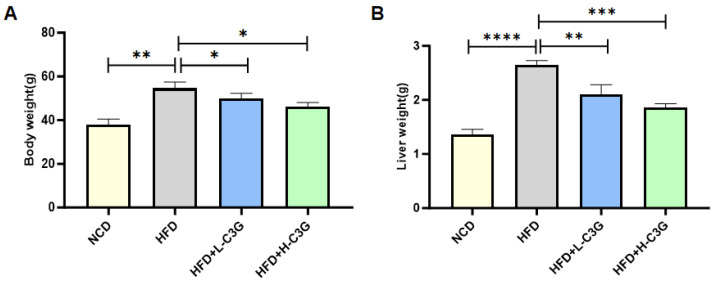
Effects of C3G on body weight and liver mass in NAFLD mice. (**A**). Effects of different treatments on body weight in mice. (**B**). Effects on liver mass. (* *p* ≤ 0.05; ** *p* ≤ 0.01; *** *p* ≤ 0.001; **** *p* ≤ 0.0001).

The results are shown in Figure 1. Compared with the NCD group, the body mass (*p* ≤ 0.01) and liver mass (*p* ≤ 0.0001) of mice in the HFD group were significantly increased; compared with the HFD group, the body mass and liver mass of the L-C3G group and H-C3G group decreased (*p* ≤ 0.01); and the body mass and liver mass of mice in the H-C3G group were higher than those in the L-C3G group.

### 2.2. Effects of C3G on Liver Function and Lipid Metabolism in NAFLD Mice

**Figure 2:**
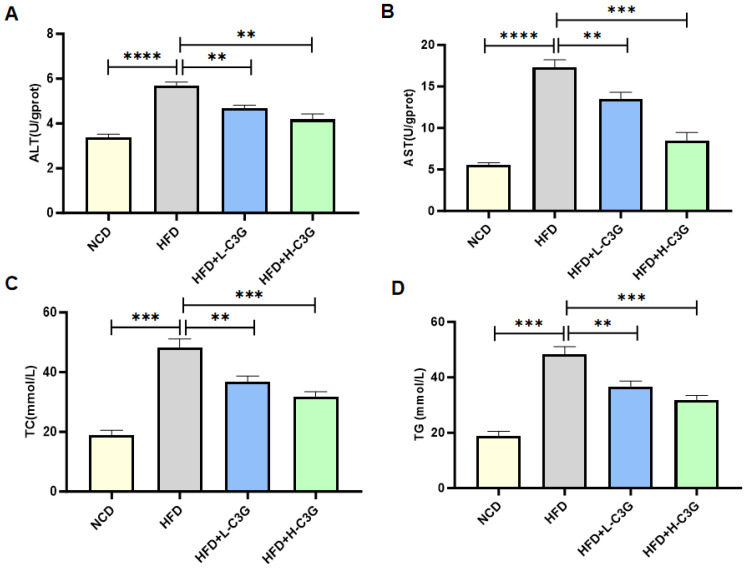
The effects of C3G on liver function and lipid metabolism in NAFLD mice. (**A**) The levels of ALT in the serum of each group of mice. (**B**) The levels of AST in the serum of mice in each group. (**C**) The levels of TC in the serum of each group of mice. (**D**) The levels of TG in the serum of each group of mice (** *p* ≤ 0.01; *** *p* ≤ 0.001; **** *p* ≤ 0.0001).

The results are shown in Figure 2. Compared with the NCD group, the serum ALT, AST, TC, and TG levels in the HFD group of mice were significantly increased (*p* ≤ 0.0001, *p* ≤ 0.001), indicating that HFD induced liver dysfunction and lipid metabolism disorders in mice, suggesting successful modeling. After treatment with L-C3G and H-C3G, serum ALT, AST, TC, and TG in mice were all reduced (*p* ≤ 0.01, *p* ≤ 0.001), with the H-C3G group showing a greater decrease.

### 2.3. Effect of C3G on Liver Pathology in NAFLD Mice

**Figure 3:**
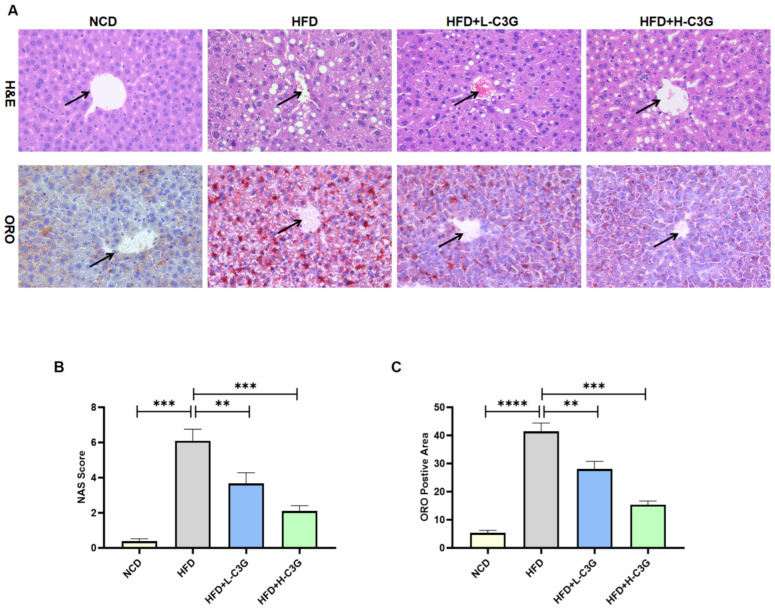
The effect of C3G on liver histopathology and lipid accumulation in NAFLD mice. (**A**) Representative pathological images of H&E staining and ORO staining of liver tissues from each group, magnification = 200×. (**B**) Quantitative analysis of NAFLD Activity Score (NAS) based on H&E staining. (**C**) Quantitative analysis of ORO-positive area in liver sections. The black arrow indicates the lesion location. (** *p* ≤ 0.01; *** *p* ≤ 0.001; **** *p* ≤ 0.0001).

We observed the pathological changes in liver tissue of each treatment group using the HE staining method, and the results are shown in Figure 3. The liver tissue structure of the NCD group mice was normal, and the liver cell structure was intact and clear, with no obvious lipid droplet deposition inside the cells; the liver tissue of HFD group mice is filled with a large number of fat vacuoles of different sizes, and the liver cell structure is disordered and swollen, with a large number of inflammatory cells infiltrating, indicating successful modeling. Comparing the liver tissues of mice treated with different doses of C3G with the NCD group, mild to moderate hepatic steatosis was still observed. However, compared with the HFD group, the degree of steatosis was reduced, the number of intracellular lipid droplets was significantly reduced, and the cell volume decreased, leading to a decrease in the infiltration of inflammatory cells.

The severity of fat accumulation can be visually reflected through Oil Red O staining, as shown in Figure 3 below. Compared with the NCD group, the HFD group showed pathological changes with significant lipid deposition; compared with the HFD group, the lipid deposition in the L-C3G and H-C3G groups was reduced.

### 2.4. Effect of C3G on Hepatic Inflammation in NAFLD Mice

**Figure 4:**
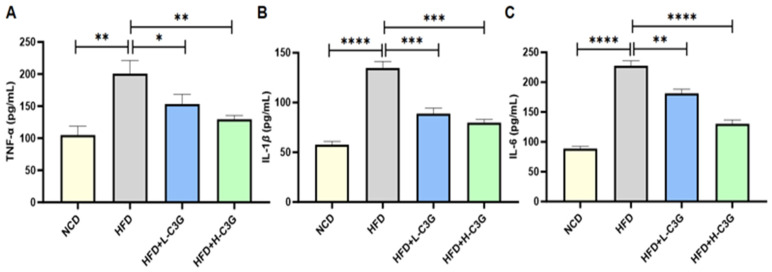
Effect of C3G on serum levels of inflammatory factors in mice fed a HFD. (**A**) TNF-α levels in mice across all groups. (**B**) IL-1β levels in mice across all groups. (**C**) IL-6 levels in mice across all groups. (* *p* ≤ 0.05; ** *p* ≤ 0.01; *** *p* ≤ 0.001; **** *p* ≤ 0.0001).

The levels of inflammatory factors in the liver tissues of each treatment group were detected using the ELISA method, and the results are shown in Figure 4. Compared with the NCD group, the levels of TNF-α, IL-1β, and IL-6 in the liver homogenate of the HFD group were significantly increased (*p* ≤ 0.01, *p* ≤ 0.0001). Different doses of C3G treatment can significantly inhibit the expression levels of TNF-α, IL-1β, and IL-6 (*p* ≤ 0.05, *p* ≤ 0.01, *p* ≤ 0.001, *p* ≤ 0.0001). Among them, compared with the L-C3G group, the H-C3G group can more significantly inhibit the levels of TNF-α, IL-1β, and IL-6.

### 2.5. Effect of C3G on the Indicators of Liver Oxidative Stress in NAFLD Mice

**Figure 5:**
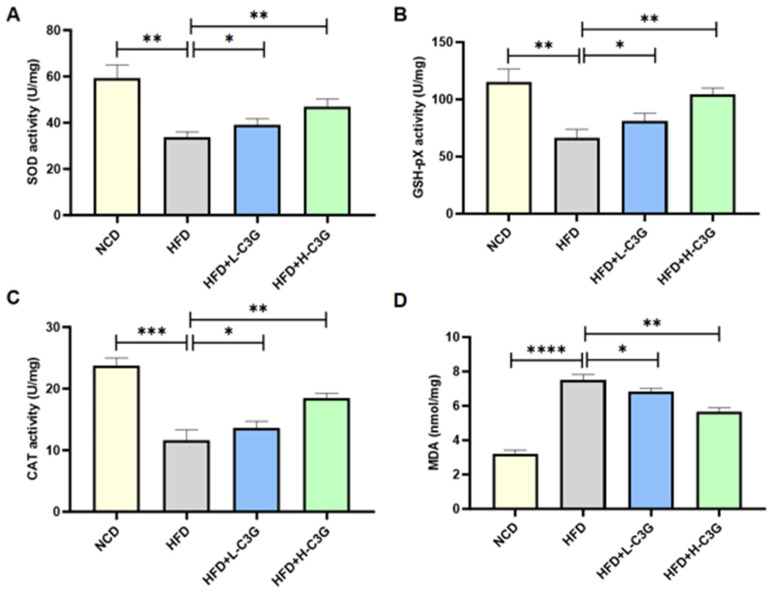
The influence of C3G on oxidative stress indicators in the livers of NAFLD mice. (**A**) The activity of SOD in each group of mice; (**B**) The activity of GSH-Px in each group of mice; (**C**) CAT activity in each group of mice; (**D**) MDA levels in each group of mice. (* *p* ≤ 0.05; ** *p* ≤ 0.01; *** *p* ≤ 0.001; **** *p* ≤ 0.0001).

We tested the liver oxidative stress capacity of each treatment group, and the results are shown in Figure 5. Compared with the NCD group, the activities of SOD, GSH-pX, and CAT in the liver tissue of the HFD group mice were significantly reduced (*p* ≤ 0.01, *p* ≤ 0.001), but the level of MDA was significantly increased (*p* ≤ 0.0001). Compared with the HFD group, different doses of C3G treatment groups significantly increased the activities of SOD, GSH-pX, and CAT in liver tissue (*p* ≤ 0.01) and decreased the levels of MDA (*p* ≤ 0.05, *p* ≤ 0.01), with the H-C3G group showing more significant changes in mice.

### 2.6. Effect of C3G on the Expression of AMPK Pathway Proteins in the Liver of NAFLD Mice

**Figure 6:**
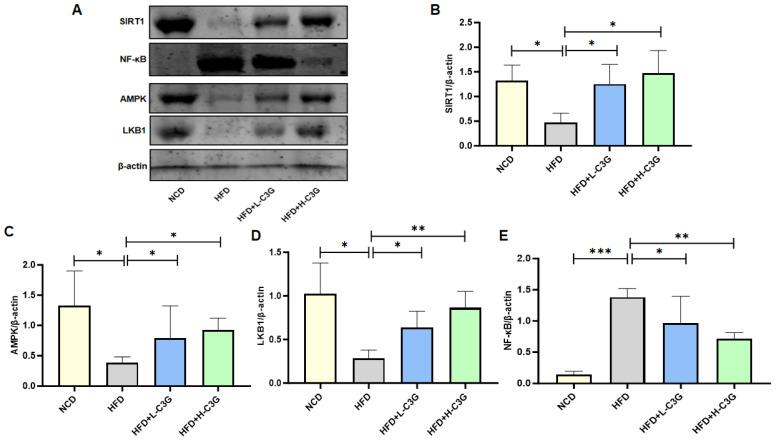
Western blot analysis of protein expression levels in the AMPK pathway. (**A**) Representative graphs of protein expression levels. (**B**) SIRT1 grayscale quantitative analysis. (**C**) AMPK grayscale quantitative analysis. (**D**) LKB1 grayscale quantitative analysis. (**E**) NF-κB grayscale quantitative analysis. (* *p* ≤ 0.05; ** *p* ≤ 0.01; *** *p* ≤ 0.001).

The expression levels of AMPK pathway proteins in liver tissue were detected using a Western blot assay, and the results are shown in Figure 6A. Compared with the NCD group, the expression of AMPK, SIRT1, LKB1, and NF-κB proteins in the liver of HFD group mice was inhibited (*p* ≤ 0.05); compared with the HFD group, C3G treatment significantly upregulated the expression of three proteins in the mouse liver (*p* ≤ 0.05, *p* ≤ 0.01), with the H-C3G group showing more significant changes.

### 2.7. Effect of C3G on NF-κB Protein in Liver of NAFLD Mice

**Figure 7:**
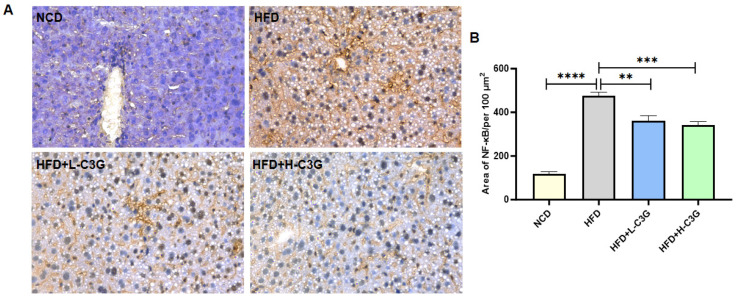
The effect of C3G on the expression of NF-κB protein in the liver tissues of NAFLD mice. (**A**) Representative images of immunohistochemical staining, magnification = 400×. (**B**) Quantitative statistical analysis of NF-κB expression regions in mouse tissues. (** *p* ≤ 0.01; *** *p* ≤ 0.001; **** *p* ≤ 0.0001).

The expression of NF-κB protein in mouse liver was detected by the IHC method, and the positive protein showed a dark brown color, as shown in Figure 7. The positive protein in the liver of the HFD group was significantly higher than that of the NCD group (*p* ≤ 0.0001), indicating an increase in the expression level of NF-κB; compared with the HFD group, the liver tissues of mice in the low and high dose C3G groups showed a decrease in brown area (*p* ≤ 0.01, *p* ≤ 0.001), indicating a decrease in the expression of NF-κB.

### 2.8. Effect of C3G on mRNA Transcription Levels of the AMPK Pathway in the Liver of NAFLD Mice

**Figure 8:**
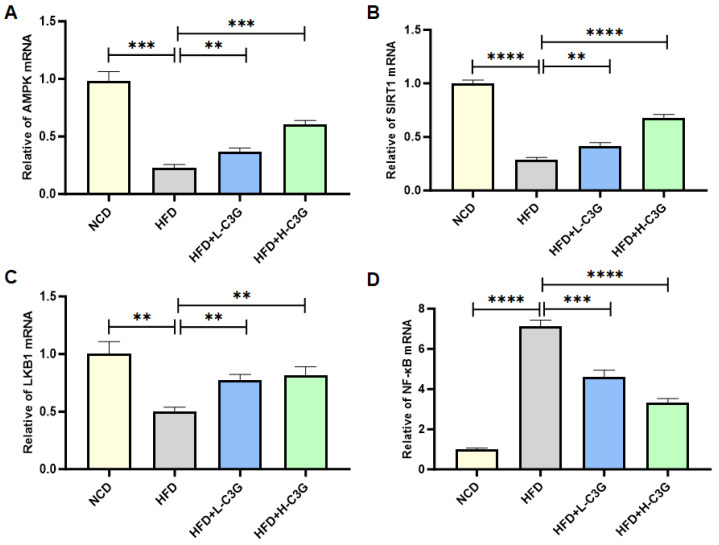
The effect of C3G on the mRNA transcription levels of the AMPK pathway in NAFLD mice. (**A**) Relative expression levels of AMPK mRNA. (**B**) Relative expression levels of SIRT1 mRNA. (**C**) Relative expression levels of LKB1 mRNA. (**D**) Relative expression levels of NF-κB mRNA. (** *p* ≤ 0.01; *** *p* ≤ 0.001; **** *p* ≤ 0.0001).

The expression of key genes in the AMPK pathway related to liver fat production and hepatic fatty acid β—oxidation metabolism was analyzed using the qPCR method, and the results are shown in Figure 8. Compared with the NCD group, the relative mRNA expression levels of AMPK, SIRT1, and LKB1 genes in the liver of the HFD group were significantly downregulated (*p* ≤ 0.001, *p* ≤ 0.0001, *p* ≤ 0.01), and the relative expression level of NF-κB gene mRNA was significantly upregulated (*p* ≤ 0.0001), indicating that the AMPK pathway in the liver of NAFLD mice was significantly inhibited. Compared with the HFD group, the relative expression levels of AMPK, SIRT1, and LKB1 mRNA were significantly upregulated in the L-C3G group (*p* ≤ 0.01) and H-C3G group (*p* ≤ 0.001, *p* ≤ 0.0001, *p* ≤ 0.01), while the relative expression level of NF-κB mRNA was significantly downregulated (*p* ≤ 0.0001).

## 3. Discussion

As an emerging functional fruit, indigo fruit has a significantly higher C3G content than traditional berries such as blueberries and wolfberries, and has attracted widespread attention in recent years. C3G is one of the main active components of anthocyanins in indigo fruit, with significant biological activities such as antioxidant, anti-inflammatory, metabolic regulation, and neuroprotection. In recent years, it has become a hot molecule in the field of natural product research [19,20,21]. Epidemiological studies have shown that non-alcoholic fatty liver disease has become the most common chronic liver disease worldwide, with a continuous increase in the prevalence of the disease and a serious threat to public health. It can gradually progress to fatty hepatitis, liver fibrosis, and even cirrhosis, causing a heavy disease burden [22]. In clinical evaluation, ALT and AST are commonly used biomarkers to evaluate liver function, while TC and TG are commonly used biomarkers to evaluate liver lipid metabolism abnormalities. This study used an HFD-induced NAFLD mouse model to investigate the improvement effect of C3G on the pathological process of liver injury in NAFLD mice. As shown in Figure 2, we found that supplementing C3G can reduce ALT and AST activity and TC and TG levels in HFD-induced NAFLD mice, indicating that C3G can restore liver function and improve liver lipid metabolism abnormalities in NAFLD mice, and is dose-dependent. Existing research has mostly focused on the role of natural flavonoids in improving lipid metabolism. The results of this study are consistent with previous reports, confirming the protective effect of C3G on NAFLD. In addition, as shown in Figure 4, we found that C3G treatment alleviates liver steatosis and pathological damage in NAFLD mice, and inhibit the expression levels of inflammatory factors TNF-α, IL-1β, and IL-6 to exert anti-inflammatory effects. There are relevant research reports that C3G can exert anti-inflammatory effects by downregulating the expression levels of pro-inflammatory factors such as IL-1β and IL-18 in NAFLD mice [19]. Zhou et al. found that C3G can inhibit the interaction between TXNIP-NLRP3, block inflammasome assembly and caspase-1 activation, reduce the levels of IL-1β and IL-18 in liver tissue of HFD-induced NAFLD mice, and alleviate pre-fibrotic inflammation [23]. In addition, C3G can improve the pathological changes in alcoholic liver injury in mice by restoring liver function, inhibiting liver lipid accumulation, and regulating intestinal microbiota [24]. The above studies all indicate that C3G has significant anti-inflammatory activity and can improve liver injury pathology, which is consistent with the results of this study.

During the occurrence and development of NAFLD, lipid accumulation in the liver can cause an imbalance in the utilization and clearance of lipids in the body, exacerbating oxidative stress and lipid peroxidation, and leading to severe inflammatory reactions [25]. When evaluating oxidative stress response, SOD, GSH-Px, and MDA are the three main measurement indicators [26]. As shown in Figure 5, the results of this study indicate that C3G treatment can alleviate oxidative stress levels in the liver of NAFLD mice, increase SOD, GSH-Px, and CAT activities, and reduce MDA content in the liver. This is consistent with the research results of Tsuda T et al., where C3G can inhibit lipid peroxidation after liver ischemia-reperfusion injury, increase GSH levels in liver tissue, and significantly alleviate oxidative stress-related liver injury [27]. In summary, C3G has significant antioxidant effects, which can enhance the body’s antioxidant capacity and alleviate inflammatory reactions.

In the molecular mechanism of NAFLD treatment, AMPK is an enzyme that controls systemic energy metabolism and is also an important pathway for targeted regulation of liver lipid metabolism. Activation of this pathway can inhibit fatty acid synthesis, activate fatty acid oxidation, alleviate liver lipid accumulation and insulin resistance, and alleviate the development of NAFLD [28]. AMPK is an energy metabolism switch associated with positive lipid regulation in the liver. Its activation can block the nuclear translocation of SREBP1c and alleviate abnormal fat production, inhibiting lipid synthesis [29,30]. SIRT1 binds to non-histone proteins such as LKB1 and peroxisome proliferator-activated receptor gamma (PPAR-γ) in the nucleus. Acetylated substrates participate in gene expression regulation of inflammation, oxidative stress, lipid metabolism, and cell apoptosis [31]. AMPK can phosphorylate SREBP1c precursor and prevent its maturation. In addition, AMPK can indirectly promote the deacetylation of LKB1 by upregulating SIRT1, thereby enhancing AMPK pathway activity, inhibiting lipid synthesis and adipogenesis, reducing NF-κB activation levels, and alleviating inflammatory responses [32]. To further investigate whether C3G improves fat production associated with NAFLD by regulating the expression of lipid metabolism-related genes, we measured the gene expression levels of the AMPK/SIRT1/NF-κB signaling pathway. As shown in Figure 8, compared to the control group, the expression levels of AMPK, SIRT1, and LKB1 genes in the livers of NAFLD mice decreased, while the expression levels of NF-κB and inflammatory factor genes increased, leading to abnormal liver function and lipid metabolism. After treatment with C3G, the expression levels of AMPK, SIRT1, and LKB1 genes in the livers of NAFLD mice increased, while the expression levels of NF-κB and inflammatory factor genes decreased, resulting in improved liver function and lipid metabolism. Park et al. found that honeysuckle extract C3G improves NAFLD pathology by activating AMPK signaling and inhibiting liver lipid metabolism gene expression, reducing triglyceride accumulation [33]. Du’s research has shown that C3G activates key factors in the AMPK pathway, enhances renal antioxidant levels, and alleviates pathological ischemia-reperfusion kidney injury [34]. All of the above are consistent with the results of this study, suggesting that C3G may be involved in the regulation of AMPK-related signaling pathways, but its molecular mechanism needs further investigation.

A key novelty of this study lies in its systematic investigation of C3G’s effects on both lipid metabolism, oxidative stress, and inflammation in an early NAFLD model, linking these effects to changes in the AMPK/SIRT1/NF-κB pathway. However, the study still has several limitations: first, the existing evidence is based solely on correlation analysis of gene expression levels and has not been directly validated through functional experiments involving pathway activation/inhibition; second, the study focused exclusively on the AMPK/SIRT1/NF-κB pathway without examining other potential signaling cascades involved in NAFLD progression, thus leaving open the possibility that C3G may target other mechanisms or pathways; third, the findings are derived from a high-fat diet-induced mouse NAFLD model, and given differences in metabolic regulatory mechanisms across species, caution is warranted when extrapolating these results to clinical applications in humans. Overcoming these limitations in future research—by conducting pathway functional validation, multi-pathway cross-analysis, and more clinically relevant model validations—would help more definitively assess the therapeutic potential of C3G for NAFLD.

## 4. Materials and Methods

### 4.1. Experimental Animal

Six-week-old male SPF mice, with a body weight of 35 ± 2 g, were purchased from Beijing Weitonglihua Experimental Animal Technology Co., Ltd. (Beijing, China), with animal license number SCXR (Jing) 2016–0006. Approved by the Medical Laboratory Animal Management Committee, the experimental animals were raised under conditions of temperature (26 ± 3) °C, humidity (55 ± 5)%, and 12 h cycles of light and darkness, with free access to water and food, while closely monitoring their health status. The feed was purchased from Beijing Huafukang Biotechnology Co., Ltd. (Beijing, China), including high-fat feed (TD10885, 45% calories from fat, containing 0.2% (*w*/*w*) total cholesterol) and standard feed (D12450B, 10.0% kcal from fat), AIN93M). The production license number is (2019) 0008. All mice were given regular feed, a pure water-free diet, and adaptive feeding for one week before conducting the experiment.

Mice were intraperitoneally anesthetized with 3% pentobarbital sodium, and the anesthetic dosage was strictly controlled to guarantee a painless condition and avoid adverse reactions or accidental death induced by overdose. Following eyeball enucleation and blood collection, the mice were humanely euthanized to reduce animal suffering. Subsequently, body weight and liver weight were measured and recorded, and blood and liver tissue samples were collected for subsequent experimental detection.

### 4.2. Animal Grouping

After one week of adaptive feeding, 6-week-old SPF mice were randomly divided into four groups, with five mice in each group: normal diet (NCD) group, high-fat diet (HFD) group, HFD+low-dose group (HFD+L-C3G, 100 mg/kg/d), and HFD+high-dose drug group (HFD+H-C3G, 200 mg/kg/d). The selection of the C3G dose was based on the results of preliminary experiments and relevant studies reported [24]. This study selected a 4-week high-fat diet intervention as the modeling period, which is a classic induction method for NAFLD mouse models [35]. Except for the NCD group, all other groups were continuously fed with an HFD (containing 60% kcal from fat, primarily lard) for 4 weeks to establish a mouse model of early NAFLD. The successful modeling was verified by monitoring body weight gain, liver weight, and body weight ratio, as described in the Results section. Starting from 5 weeks, the low and high dose C3G groups were administered with 100 and 200 mg/kg C3G for 4 consecutive weeks, respectively. At the same time, the NCD and HFD groups were orally administered with distilled water of equal volume. After the last administration, mice in each group were fasted for 12 h. After enucleation and blood collection, the mice were euthanized, and their body weight and liver weight were recorded. Blood and liver samples were collected for testing.

### 4.3. Serological Marker Testing

The blood was placed in a 1.5 mL centrifuge tube and allowed to stand on ice for 30 min. It was then centrifuged at 3200 r/min for 10 min. The supernatant was collected and the serum levels of ALT, AST, Total Cholesterol (TC), and TG were measured using the microplate method with the Aspartate Aminotransferase (AST) kit (MAK055, Sigma, St. Louis, MO, USA), Alanine Aminotransferase (ALT) kit (MAK052, Sigma, St. Louis, MO, USA), Cholesterol kit (MAK043, Sigma, St. Louis, MO, USA), and Triglyceride (TG) kit (MAK266, Sigma, St. Louis, MO, USA). Please refer to the instructions of the reagent kit for specific testing methods. To eliminate protein differences between samples and ensure data reliability, all test results are normalized and corrected based on total protein concentration. Standardizing hormone levels to total protein can avoid serum concentration deviations caused by factors such as blood collection volume, sample processing, and hemolysis, effectively improving the accuracy and comparability of inter-group data.

### 4.4. Liver Pathological Observation

Fix liver tissue with paraformaldehyde solution, embed in paraffin, prepare 6 μm thick paraffin sections, perform HE staining according to the instructions of the HE kit (ab245880, Abcam, Cambridge, UK), and seal the sections with neutral gum; another OCT was used to embed liver tissue, and 6 μm thick sections were prepared. Oil red O staining solution (O0625, Sigma, St. Louis, MO, USA) was used for 20 min and washed with PBS. Finally, Mayer hematoxylin was added to stain the cell nucleus for 1–2 min and washed with PBS. Observe the stained tissue under an optical microscope(Carl Zeiss Microscopy GmbH, Jena, Germany).

### 4.5. Detection of Inflammatory Factor Levels

Weigh 0.5 g of mouse liver tissue, add 4.5 mL of physiological saline to a glass homogenizer tube, grind until homogenized, centrifuge at 3000 r/min for 10 min at 4 °C, retain the supernatant, and obtain 10% mass fraction of liver homogenate. Adopt the tumor necrosis factor-α (TNF-α) kit (E-HSEL-R0001, Elabscience, Wuhan, China), interleukin-1β (IL-1β) kit (E-EL-R0012, Elabscience, Wuhan, China), and interleukin-6 (IL-6) kit (900-K86, Thermo Fisher, Waltham, MA, USA). The operating instructions for the reagent kit are to detect the levels of liver inflammatory factors TNF-α, IL-1β, and IL-6 separately.

### 4.6. Detection of Oxidative Stress Indicators in Liver Tissue

Prepare 10% liver homogenate using the same method as Section 4.5, and then proceed according to the superoxide dismutase (SOD) kit (BC5130, Solarbio, Beijing, China), glutathione peroxidase (GSH-Px) kit (BC1195, Solarbio, Beijing, China), catalase (CAT) kit (BC0205, Solarbio, Beijing, China), and malondialdehyde (MDA) kit (BC6415, Solarbio, Beijing, China). Instructions for operation: detect the levels of SOD, GSH-Px, MDA, and CAT in liver tissue.

### 4.7. Western Blotting Analysis

Western blotting analysis was used to detect the expression levels of AMPK pathway proteins in liver tissue. Extract liver tissue proteins from each group using a total protein extraction kit, and detect the concentration of extracted proteins using the BCA method. After electrophoresis and membrane transfer, place it in skim milk powder and refrigerate at 4 °C overnight. On the second day, the membranes were rinsed with TBST buffer, followed by incubation with primary antibodies against AMPK (1:1000, ab133448, Abcam, Cambridge, UK), SIRT1 (1:1000, ab110304, Abcam, Cambridge, UK), LKB1 (1:1000, ab199970, Abcam, Cambridge, UK), NF-κB (1:1000, ab194908, Abcam, Cambridge, UK) and β-actin (1:1200, ab8226, Abcam, Cambridge, UK) for 1 h at room temperature. The membranes were then washed again with TBST buffer. Finally, the membrane was scanned and detected using the Odyssey DLx dual color infrared laser imaging system.

### 4.8. q-PCR

Total RNA was extracted from the liver tissues of mice in each treatment group using an RNA extraction kit (R1200, Solarbio, Beijing, China), and the RNA was reverse transcribed into cDNA according to the instructions of the reverse transcription kit. Primer sequence design and synthesis were carried out based on key genes in the AMPK/SIRT1/NF-κB signaling pathway, and q-PCR was used for mRNA transcription level detection, with β-actin selected as the internal reference gene. The primer sequences are shown in Table 1. Amplification efficiency was verified, and relative expression was computed using the 2^−∆∆CT^ method [36].

### 4.9. Immunohistochemical Detection of NF-κB Protein in Liver Tissue

Using immunohistochemistry (IHC), liver tissue sections were sequentially subjected to xylene dewaxing, citric acid anti-stock solution repair, catalase incubation, addition of NF-κB antibody (1:400, ab16502, Abcam, Cambridge, UK) at 37 °C for 1 h, 3-diaminobenzidine staining, and xylene transparency. After completion, neutral gum was used to seal the sections, and images were observed and collected under an optical microscope.

### 4.10. Statistical Analysis

All data are presented as mean ± standard deviation (x ± s) and analyzed for one-way ANOVA using SPSS 28.0.1.1 statistical software. Duncan’s multiple range test was selected as the post hoc test for subsequent pairwise comparisons due to its good applicability in multi-group comparison and stable control of the type I error rate. GraphPad Prism 9.5 software was used for plotting, and statistical significance was expressed as * *p* ≤ 0.05, indicating significance; ** *p* ≤ 0.01, highly significant; *** *p* ≤ 0.001, extremely significant; **** *p* ≤ 0.0001; ns, there is no significant difference.

## 5. Conclusions

The results of this study indicate that C3G intervention can alleviate liver pathological damage, improve liver function and lipid metabolism disorders, enhance liver antioxidant capacity, and reduce inflammatory response in NAFLD mouse models. It should be noted that the gene and protein expression changes observed in this study suggest that the AMPK/SIRT1/NF-κB pathway may be involved in mediating the liver protective effect of C3G, but further functional experiments are needed to confirm the direct activation evidence of this pathway. This research provides experimental evidence for further developing C3G as a potential therapeutic drug for non-alcoholic fatty liver-related liver injury.

## Figures and Tables

**Table 1:** Sequence of target gene primers.

Name	Sequences (5′-3′)
AMPK	AATTCCGCCTACCTCTGCACT
	GGAGGAAGAAGGTCTTCGG
SIRT1	TGATTGGCACCGATCCTCG
	CCACAGCGTCATATCATCCAG
LKB1	GATAGAGCGCAACAAGCAGAA
	CCTTGCCGTAAGAGCCTTCC
NF-κB	GACGATCTGTTTCCCCTCAT
	GCTTCTCTCCCCAGGAATAC
β-actin	CCTGCGGCATTCACGAAACTAC
	ACTCCTGCTTGCTGATCCACAATC

## Data Availability

The original contributions presented in the study are included in the article. Further inquiries can be directed to the corresponding authors.

## Abbreviations

The following abbreviations are used in this manuscript:

C3G	Cyanidin-3-O-Glucoside
NAFLD	Non-Alcoholic Fatty Liver Disease
NCD	Normal diet
HFD	High-fat diet
AMPK	Adenylate-Metabolizing Protein Kinase
SIRT1	Silencing Information Regulator 1
NF-κB	Nuclear Factor κB
LKB1	Liver Kinase B1
SOD	Superoxide dismutase
GSH-Px	Glutathione peroxidase
CAT	Catalase
MDA	Malondialdehyde
ALT	Alanine aminotransferase
AST	Aspartate aminotransferase
TC	Total cholesterol
TG	Triglycerides
